# Agreement and reliability of hepatic transient elastography in patients with chronic hepatitis C: A cross‐sectional test–retest study

**DOI:** 10.1002/hsr2.1184

**Published:** 2023-04-03

**Authors:** Oskar Ljungquist, Jon Olinder, Jonas Tverring, Charlott Kjölvmark, Gustav Torisson

**Affiliations:** ^1^ Department of Infectious Diseases Helsingborg Hospital Helsingborg Sweden; ^2^ Division of Infection Medicine, Department of Clinical Sciences Lund University Lund Sweden; ^3^ Clinical Infection Medicine, Department of Translational Medicine, Faculty of Medicine Lund University Malmö Sweden; ^4^ Department of Infectious Diseases Skåne University Hospital Malmö Sweden

**Keywords:** agreement, hepatitis C, interobserver, reliability, transient elastography

## Abstract

**Background and Aims:**

Transient elastography (TE) has largely replaced liver biopsy to evaluate fibrosis stage and cirrhosis in chronic hepatitis C. Previous studies have reported excellent reliability of TE but agreement metrics have not been reported. This study aimed to assess interrater agreement and reliability of repeated TE measurements.

**Methods:**

Two operators performed TE independently, directly after each other. The primary outcome was disagreement, defined as a difference in TE results between operators of ≥33%, as well as the smallest detectable change, SDC_95_ (i.e., the difference between measurements needed to state with 95% certainty that there is a difference in underlying stiffness). Secondary outcomes included reliability, measured as intraclass correlation (ICC), and patient and examination characteristics associated with the agreement.

**Results:**

In total, 65 patients were included, with a mean liver stiffness of 9.7 kPa. Of these, 21 (32%) had a disagreement in TE results of ≥33% between the two operators. The SDC_95_ on the log scale was 1.97, indicating that an almost twofold increase or decrease in liver stiffness would be required to confidently represent a change in the underlying fibrosis. Reliability, estimated using the ICC, was acceptable at 0.86. In a post hoc analysis, fasting less than 5 h before TE was associated with a higher degree of disagreement (48% vs. 19%, *p* = 0.03).

**Conclusions:**

In our clinical setting, interrater agreement in directly repeated TE measurements was surprisingly low. It is essential to further investigate the reliability and agreement of TE to determine its validity and usefulness.

## 1 INTRODUCTION

Early identification of fibrosis and cirrhosis of the liver enables prevention and treatment of long‐term consequences of chronic hepatitis C, including liver failure, hepatocellular cancer, and death.^1^ Even where antiviral treatment is generally offered, fibrosis and cirrhosis staging affect decisions regarding surveillance, for hepatocellular cancer and esophageal varices.^2^ Traditionally, liver biopsy has been the reference standard of fibrosis and cirrhosis assessment, despite being associated with discomfort and complications.^3^ Transient elastography (TE) is a noninvasive method for measuring liver stiffness that has replaced liver biopsy in a majority of patients with chronic hepatitis C.^4^ Due to its noninvasive nature, TE is also suitable for longitudinal monitoring.

Previous studies suggest comparable accuracy between liver biopsy and TE to detect fibrosis in hepatitis C patients.^5^, ^6^ Studies have reported excellent test–retest reliability for TE.^7^, ^8^, ^9^, ^10^, ^11^ Reliability refers to an instrument's ability to discriminate between study subjects and may be excellent even with considerable measurement error if the population is heterogenous.^12^, ^13^, ^14^, ^15^ Agreement, in contrast, refers to differences in measurements on the original scale (kPa for TE). Whether the agreement is acceptable is a situation‐dependent clinical consideration and can be related to the smallest detectable change (SDC).^13^, ^16^ SDC corresponds to the following clinical question: if a patient has performed TE previously, how much higher, or lower, must a new TE result be to confidently represent a change in underlying fibrosis? This question cannot be addressed using reliability statistics alone and, to the best of our knowledge, agreement metrics or SDC have not been addressed in previous studies.

The aim of the present study was to assess agreement, SDC, and reliability of repeated measurements of liver stiffness of TE in chronic hepatitis C patients, and to explore factors associated with disagreement.

## 2 METHODS

### 2.1 Study setting and patients

This was a cross‐sectional observational study, conducted at the outpatient clinic of the infectious diseases department of Helsingborg Hospital, in the south of Sweden, serving a population of approximately 250,000 inhabitants. Patients were recruited by convenience sampling between November 2018 to February 2022. Eligibility criteria were nonpregnant adult (≥18 years) patients with chronic hepatitis C, as defined by at least one positive serum hepatitis C virus (HCV)‐RNA and no evidence of acute hepatitis C. Thus, exclusion criteria were pregnancy and acute hepatitis C. Data on age, sex, date, operators, the probe used (medium or extra large [XL]), stiffness (kPa), time since last intake of food (hours), time since last intake of alcohol (days), length (cm), weight (kg), HCV genotype, and liver biomarkers were recorded.

### 2.2 TE procedures

Liver stiffness was measured in kPa using the Fibroscan® device (Echosens), for which the reported kPa value was the median of 10 consecutive measurements. The interquartile range divided by the median (IQR %) was noted. The TE was considered valid when 10 consecutive successful measurements had been gathered, and the IQR % was lower than 30%, as in the clinical routine. The Fibroscan® was operated by a total of nine individuals: eight medical doctors and one specially trained nurse. The majority (*n* = 6) of the medical doctors were specialists in infectious diseases, and two were resident physicians. All operators had been certified by the company behind Fibroscan®, but none of the operators were experienced operators, as all had performed less than 500 exams. The TE experience of each observer is detailed in Supporting Information: Table S4.

Patients were put in the recommended body position, with the right arm on top of the head and legs positioned towards the left. This body position was unaltered during the entire procedure. Patients were assessed twice by each operator, with the probe removed between examinations. The second operator assessed the patients directly after the first, without intermission. Operator one and two performed the measurements independently, and operator two were unaware of the results from the previously performed TE. The probe position for the previous measurement was not known but typical marks generated by the probe on the patient remained. Operators could select a medium or XL probe at their own discretion based on the patient's configuration.

### 2.3 Categorization of liver stiffness scores

Continuous scores in kPa were categorized into stages (*F* scores) using cutoff values suggested by Castera: F0/F1 ≤ 7.1 kPa, F2 > 7.1 and <9.5, F3 ≥ 9.5 and <12.5, and F4 (cirrhosis) ≥ 12.5.^17^ We further prespecified a dichotomous cutoff where a difference in liver stiffness between operator one and two of ≥33% was considered unacceptably high. In a worst‐case scenario, this could correspond to a two step‐change in *F* score staging (e.g., 9.4 kPa (F2) × 1.33 = 12.5 kPa (F4)).

### 2.4 Outcomes

The primary outcome was disagreement, expressed as (1) the proportion of participants with interrater differences at or above our prespecified threshold (33%) and (2) the SDC, representing the difference needed to state with 95% certainty that a change had occurred in the underlying fibrosis (SDC_95_). Reliability was the secondary outcome, for continuous stiffness measurements as well as for fibrosis stage categories.^18^ As an exploratory outcome, patient and exam characteristics associated with interrater differences above our prespecified threshold of ≥33% in liver stiffness were evaluated.

### 2.5 Statistics

#### 2.5.1 Agreement

The first rating for each operator was used for interrater analysis. We estimated the 95% limits of agreement (LOA_95_, the range encompassing 95% of differences), as described by Bland and Altman.^16^ We expected heteroscedasticity (i.e., larger differences at the higher end of the scale) and measurements were transformed using the natural logarithm. The standard error of measurement (SEM) was estimated on the log scale and SDC_95_ was derived from SEM.^13^

#### 2.5.2 Reliability

A one‐way random effects intraclass correlation (ICC_1, 1_) was used to estimate reliability in continuous kPa.^13^, ^16^ Cohen's κ was evaluated for the *F* score as well as separately for the dichotomous outcome of F0–3 versus F4 (cirrhosis no vs. yes). ICC and *κ* values are expressed with 95% confidence intervals (CIs) within brackets. Intrarater agreement and reliability were determined using the same methods.

#### 2.5.3 Factors associated with discordance in liver stiffness

The other baseline variables were explored for associations with the dichotomous disagreement outcome, using nonparametric tests throughout.

## 3 RESULTS

### 3.1 Participant and liver stiffness characteristics

In total, 66 patients were asked to participate, of which one declined, and 65 were included. In 60 patients, four (2 + 2) valid measurements were obtained by the two operators, and in 5 patients, three (2 + 1) measurements were obtained (in 1 patient, one measurement was invalid, and in 4 patients, the second rating was not performed). There were 255 examinations performed overall. For interrater analysis, 130 measurements were used (65 × 2) and for intrarater analysis, 250 measurements (125 × 2) were used. The patient and examination characteristics are displayed in Table 1 and Table 2, respectively.

**Table 1:** Patient characteristics of included patients.

Patient characteristics	
Age	46 (35–57)
Male sex	51 (78%)
Time since food intake, h (*n* = 62)	5 (2–12)
Time since alcohol intake, days (*n* = 63)	30 (4–182)
Body mass index, kg/m^2^ (*n* = 62)	25 (22–29)
HCV genotype	
1a	24 (37%)
1b	4 (6%)
2b	7 (11%)
2c	1 (2%)
3a	29 (45%)
HCV‐RNA, million copies (*n* = 64)	2 (0–5)
ALT, µkat/L (*n* = 64)	1.4 (0.8–2.2)
AST, µkat/L (*n* = 58)	0.9 (0.6–2.1)
Albumin, g/L (*n* = 54)	41 (38–43)
Platelets, count (*n* = 59)	246 (197–291)
Bilirubin, µmol/L (*n* = 50)	8 (6–11)
ALP, µkat/L (*n* = 57)	1.4 (1.1–1.7)
GT, µkat/L (*n* = 54)	1 (0.6–2.2)
PK‐INR (*n* = 59)	1 (1–1.1)

*Note*: Continuous variables are expressed as median (IQR), categorical as *n* (%), *n* = 65 unless otherwise specified.

Abbreviations: ALP, alkaline phosphatase; ALT, alanine transaminase; AST, aspartate aminotransferase; GT, gamma‐glutamyl transferase; HCV, hepatitis C virus; IQR, interquartile range; PK‐INR, prothrombin complex concentrate.

**Table 2:** Characteristics of included exams.

Examination characteristics	
Medium probe	225 (87%)
X‐large probe	32 (12%)
Stiffness, kPa	7.3 (5.4–10.5)
IQR, %, *n* = 254	13 (9–18)
*F* score*	
F0/F1	118 (45%)
F2	63 (24%)
F3	21 (8%)
F4	53 (20%)

*Note*: Continuous variables are expressed as median (IQR), and categorical as *n* (%). *N* = 255, unless otherwise specified.

Abbreviation: IQR, interquartile range.

* *F* scores according to Castera.^17^

### 3.2 Agreement

**Figure 1:**
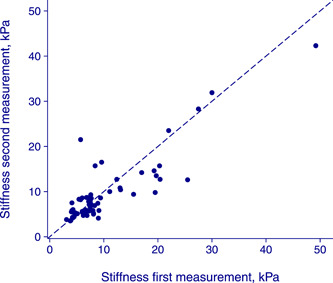
Scatterplot of LS ratings by the operator, in kPa. Each dot represents a subject with the rating according to the first operator on the *x* axis and according to the second on the *y* axis. The dotted line represents perfect agreement. LS, liver stiffness.

**Figure 2:**
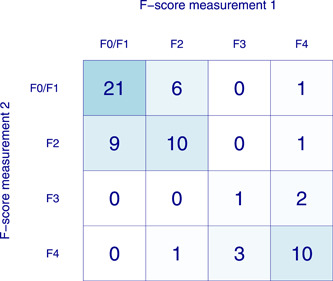
Ratings of liver stiffness by operator 1 and operator 2, in *F* scores, categorized according to Castera.^17^

**Figure 3:**
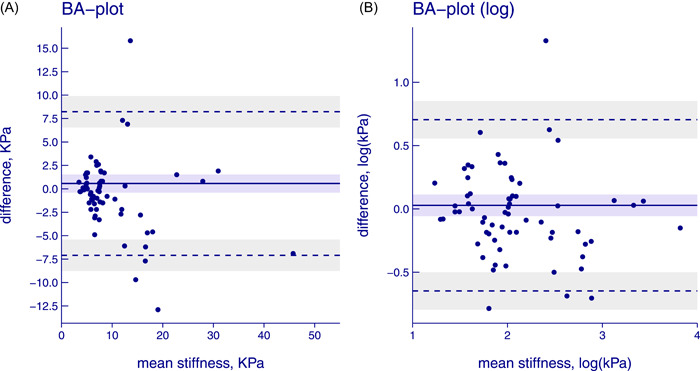
(A) BA plot of between operator. Differences on the *y* axis and mean stiffness rating of the two operators in kPa on the *x* axis. The dots represent subjects, the blue line represents bias, the dotted lines represent LOA_95_, and shaded areas have 95% confidence intervals (original scale). (B) BA plot of between operator. Differences on the *y* axis and mean stiffness rating of the two operators in kPa on the *x* axis. The dots represent subjects, the blue line represents bias, the dotted lines represent LOA_95_, and shaded areas have 95% confidence intervals (log scale). BA, Bland–Altman; LOA_95_, 95% limits of agreement.

The results of the first and second operators are displayed in Figure 1. In total, 23 patients (35%) were classified into different *F* scores by the first and second operators, of whom 8 (12% of all) had an F4 discrepancy, see Figure 2. Interrater disagreement at or above the prespecified threshold of 33% was found in 21 out of 65 patients (32%). The LOA_95_ on the original scale was 0.6 ± 7.7 kPa and Bland–Altman plots confirmed heteroscedasticity and the need for log transformation, see Figure 3A,B as well as Supporting Information: Tables S1 and S2. Furthermore, one extreme outlier affected the results and was addressed in a sensitivity analysis, see Supporting Information: Table S3. This analysis did not change results significantly and the outlier was kept in the analysis.

The SEM was estimated at 0.24 on the log scale, corresponding to an SDC_95_ of 0.68. After back‐transforming the logged values, the SDC_95_ was estimated to be 1.97. This implies that for a patient with a previous TE result of 8.0 kPa, a new result would have to be either below 4.1 kPa (8.0/1.97) or above 15.8 kPa (8.0 × 1.97) to represent a change in the underlying fibrosis with 95% certainty.

### 3.3 Reliability

Interrater reliability, estimated by ICC, was 0.86 (0.78–0.91). When liver stiffness was categorized into *F* scores, the weighted *κ* (with equal weights) was 0.66 (0.53–0.79). The corresponding *κ* value for the dichotomous comparison of F4 versus F0–3 was 0.64 (0.41–0.87).

### 3.4 Intrarater analysis

In the intrarater analysis, all metrics were better. The *F* scores were discordant in 24 of 125 (19%) and only 10 of 125 (8%) examinations had a disagreement above the prespecified threshold of ≥33%. The SDC_95_ was 1.40. For intrarater reliability, the ICC was 0.96 (0.94–0.97). After categorization, the equal‐weighted *κ* was 0.84 (95% CI: 0.77–0.90) and the dichotomous *κ* (F4 vs. F0–3) was 0.95 (0.88–1.0).

### 3.5 Factors associated with interrater disagreement

When associations with disagreement ≥33% were explored, none of the nine individual operators were under‐ or overrepresented (all operators with ≥5 examinations were involved in discordant ratings between 29% and 40%). The only factor associated with a disagreement ≥33% was the shorter duration of fasting before elastography, see Table 3. Due to this association, we performed a post hoc analysis, where interrater differences were plotted versus fasting time (see Supporting Information: Figure S5). Graphically, there seemed to be better agreement when the participant had fasted ≥5 h. We performed a subgroup analysis for long fasting (≥5 h) versus short fasting, where each group consisted of 31 of 62 participants with data on fasting time. The interrater disagreement of ≥33% was notably more common in the short fasting group (48% vs. 19%, *p* = 0.03, *χ* ^2^ test). However, there was no significant difference between groups regarding *F* score reclassification, 39% were reclassified in the short fasting group, compared to 31% in the longer fasting group, *p* = 0.72. *χ* ^2^ test.

**Table 3:** Patient and examination characteristics by interrater differences.

	Difference < 33%, *n* = 44	Difference ≥ 33%, *n* = 21	*p* Value
Age	47 (37–57)	42 (35–58)	0.88
Male sex	33 (75%)	18 (86%)	0.51
Time since food, h	7 (4–14)	3 (2–5)	**0.01**
Time since alcohol, days	15 (2–168)	60 (21–365)	0.14
BMI	25 (22–29)	26 (21–30)	0.89
HCV‐RNA, million copies	2.2 (0.5–4.9)	1.6 (0.3–5.4)	0.71
ALT, µkat/L (*n* = 64)	1.4 (0.8–2.8)	1.2 (0.7–1.8)	0.44
AST, µkat/L (*n* = 58)	1 (0.6–2.1)	0.9 (0.6–1.2)	0.39
Albumin, g/L (*n* = 54)	41 (38–43)	40 (39–42)	0.92
ALP, µkat/L (*n* = 57)	1.4 (1.2–1.7)	1.4 (1.1–1.8)	0.89
GT, µkat/L (*n* = 54)	1.1 (0.7–2.2)	0.6 (0.4–1.7)	0.20
PK‐INR (*n* = 59)	1 (1–1.1)	1 (1–1.1)	0.43
Probe change	0 (0%)	2 (10%)	0.19
IQR %, highest	16 (11–21)	17 (15–20)	0.61
HCV genotype			0.66
1a	15 (34%)	9 (43%)	
1b	2 (5%)	2 (10%)	
2b	6 (14%)	1 (5%)	
2c	1 (2%)	0 (0%)	
3a	20 (45%)	9 (43%)	

*Note*: Interrater differences have been dichotomized into below or above the prespecified threshold (33%). Continuous variables are expressed as median (IQR), and categorical as *n* (%). Bold value is statistically significant at *p* < 0.05.

Abbreviations: ALP, alkaline phosphatase; ALT, alanine transaminase; AST, aspartate aminotransferase; BMI, body mass index; GT, gamma‐glutamyl transferase; HCV, hepatitis C virus; IQR, interquartile range; PK‐INR, prothrombin complex concentrate.

## 4 DISCUSSION

This cross‐sectional study aimed to assess agreement, SDC, and reliability of repeated measurements of liver stiffness of TE in chronic hepatitis C patients, and to explore factors associated with disagreement. The interrater agreement has not been previously reported, and in our setting 32% of patients had an interrater disagreement above our prespecified threshold. Furthermore, an almost twofold increase or decrease in kPa was required to represent a change in the underlying fibrosis with 95% certainty. In a post hoc analysis, we found that longer fasting time before TE was associated with better interrater agreement.

The two TE operators found different *F* scores in 35% of participants, compared to previous studies ranging from 23% to 35%.^7^, ^8^, ^11^, ^19^ Although agreement metrics or SDC have not been presented before, a few studies provide Bland–Altman plots, from which LOA_95_ could be roughly estimated. A study by Fraquelli et al. showed LOA_95_ of bias ± approximately 4 kPa.^9^ Another study by Perazzo et al. found systematic bias between operators but also seem to display a LOA_95_ of bias ±approximately 10 kPa.^7^ Our LOA_95_ on the original scale was biased ±7.7 kPa, although heteroscedasticity (also suggested in previous studies) complicates the comparison. Thus, agreement in the current study seems to be on the lower end compared to previous studies.

Interrater reliability, measured by ICC, was 0.86 which could be considered “good to excellent” but is also on the lower end compared to previous TE studies ranging from 0.76 to 0.98.^7^, ^8^, ^9^, ^10^, ^11^, ^20^ The *κ* value of 0.64 for F4 versus F0–3 was lower compared to 0.75 and 0.80 in previous studies, but categorized values could be more sensitive if they are close to cutoffs.^7^, ^8^ Reliability measures may also be lower in a more homogenous study population. Our population had a lower median liver stiffness (LS) than in previous studies, with 15% having a unanimous F4 rating, compared to 18%–36% in previous studies, possibly indicating a more homogenous population, with lower kPa values.^7^, ^8^, ^9^, ^11^, ^19^ Reclassification is less likely in the higher range as all values above 12.5 kPa would be F4. To conclude, both larger variations as well as a more homogenous population could have affected reliability negatively.

In a post hoc analysis, an agreement was better when patients had fasted for ≥5 h. *F* score consistency was not better with longer fasting, but reclassification is population‐dependant, with values closer to cutoffs more likely to be reclassified. Previous studies on fasting time have shown that 150 min after a meal, liver stiffness has returned to baseline.^21^ However, we found that the variability seemed to be increased up to 5 h after food intake, although in a secondary analysis. Previously, high body mass index, liver biomarkers, and high IQR % have also been associated with invalid TE measurements.^22^ We did not find an association between these factors and TE interrater variability. Findings related to operator experience have been conflicting.^8^, ^22^, ^23^, ^24^ Our results did not suggest systematic differences between operators, although statistical power was insufficient for formal analysis. In two participants, the two operators used different probes (medium and XL), resulting in large kPa differences, as seen in Table 3.

Reliability and agreement metrics were much better in intrarater than interrater analysis, indicating that the change of operator introduced variability. In the intrarater situation, the operator performed two consecutive exams right after each other. The rater thus had more information, regarding probe placement, probe angle, choice of the probe, and the results from the previous exam. This is an artificial situation that does not represent clinical routine, where the same rater typically would perform exams at least a year apart. Even so, the intrarater SDC_95_ was 1.40, signaling a higher variability than the IQR % for the 10 readings of each result. This may be explained by the fact that, in the intrarater situation, the probe was removed and then replaced, in contrast to the 10 repeated measurements of each LS result. The protocol did not specify that the same probe location should be used, and previous studies suggest variability due to probe location.^25^

This study has several limitations. We used many operators, who did not have extensive experience in an international context. However, all operators were certified and are performing TE within the clinical routine. The sample size was relatively small, making it sensitive to outliers, as elaborated in the Supporting Information: Appendix. In the interrater analysis, there was information bias, as marks from the preceding procedure could be visible, which could decrease variability if the same probe location was chosen. On the other hand, the operator was blinded to the previous choice of probe (which would normally be documented), and in two cases using different probes resulted in high variability.

This is a small study, and our results need verification in other settings. As TE is increasingly being used in other diagnoses, studies in these should be emphasized as well, including in hepatitis B. In such studies, reporting both reliability and agreement should be encouraged, employing the Guidelines for Reporting Reliability and Agreement Studies guidelines.^26^ In this study, the methodology focussed on an agreement in liver stiffness on the continuous kPa scale. However, from a clinical perspective, the finding that eight patients (12%) received different F4 ratings is possibly the most important one, as this would entail different allocation to screening for hepatocellular cancer. This proportion is dependent on both the study population as well as the cutoffs used for F0–F4 classification, and another definition could lead to other results. This suggests that values close to cutoffs with clinical importance should be scrutinized, and that repeated TE and/or liver biopsy considered in selected patients. Furthermore, the wide range of SDC_95_ suggests that it may be difficult to determine whether changing TE results in longitudinal monitoring represent measurement error or progressive fibrosis. Lastly, our post hoc analysis emphasizes the importance of fasting before TE and that clinicians may consider postponing TE in the case of insufficient fasting.

## 5 CONCLUSION

In our clinical setting, agreement in repeated TE measurements was surprisingly low. It is essential to further investigate the reliability and agreement of TE to determine its validity and usefulness.

## AUTHOR CONTRIBUTIONS

**Oskar Ljungquist**: Conceptualization; funding acquisition; methodology; project administration; writing—original draft; writing—review and editing. **Jon Olinder**: Data curation; writing—review and editing. **Jonas Tverring**: Data curation; methodology; validation; writing—original draft; writing—review and editing. **Charlott Kjölvmark**: Data curation; project administration; writing—review and editing. **Gustav Torisson**: Conceptualization; formal analysis; methodology; software; supervision; validation; visualization; writing—original draft; writing—review and editing.

## CONFLICTS OF INTEREST STATEMENT

The authors declare no conflict of interest.

## ETHICS STATEMENT

The Regional Ethical Review Board in Lund, Sweden, approved the study (2018‐688). All patients included in the study signed informed consent. All methods were carried out in accordance with the declaration of Helsinki and the Guidelines for Reporting Reliability and Agreement Studies guidelines.^26^

## TRANSPARENCY STATEMENT

The lead author Oskar Ljungquist affirms that this manuscript is an honest, accurate, and transparent account of the study being reported; that no important aspects of the study have been omitted; and that any discrepancies from the study as planned (and, if relevant, registered) have been explained.

## Supporting information

Supporting information.

## Data Availability

The underlying data set cannot be publicly shared, due to ethical concerns. The underlying data may be requested by individual researchers from the corresponding author upon reasonable request if approval is granted by the Swedish Ethical Review Authority. Such requests will also be evaluated by the Data Protection Officer at Skåne University Hospital.
