# Supplementary material for: Agreement and reliability of hepatic transient elastography in patients with chronic hepatitis C: A cross‐sectional test–retest study
Source: Health Sci Rep. 2023 Apr 3;6(4):e1184. doi: 10.1002/hsr2.1184 (PMC10069238; doi:10.1002/hsr2.1184)
Supplement: Supplementary file 1 — Supporting information. [file HSR2-6-e1184-s001.docx]

**SUPPLEMENTARY APPENDIX**

**METHODS**

**AGREEMENT**

Agreement refers to the difference between two measurements and is expressed on the same scale as the measurement of interest (kPa).^1-7^ Whether agreement is “good” is a situation-dependent clinical decision and there are no universal cut-offs or rules of thumb (e.g., a scale with a measurement error of 0.5 kilograms may be acceptable in adults but not in neonates). Preferably, an “acceptable” agreement should be pre-defined according to the clinical situation at hand.

**The Bland-Altman analysis and plot**

Agreement analysis was popularized in a widely cited paper by Bland and Altman.^1^ In this analysis, the mean (bias) and standard deviation (SD_d_) of difference between measurements is determined. The original paper featured the so called “Bland - Altman plot” - where the differences between measurements is expressed on the y axis and the mean of the two measurements on the x scale. In the plot, there are three lines, one representing the bias and two representing lower and upper limits of agreement (LOA), see example below. The LOA is the range in which a certain amount (often 95%) of differences lie. The LOA_95_ can be estimated by using the formula LOA_95_ = bias ± 1.96 * SD_d_.^1-3^ The plot should be used to screen for heteroscedasticity (where the differences are larger when x is larger) and to visualize where the LOA lines are, not to determine whether agreement is good. Bland and Altman also recommend providing 95% confidence intervals for bias and LOA_95_. An example of a Bland-Altman plot is shown below:

**Figure S1**. Example of Bland-Altman plot, with bias (solid line) and LOA_95_ (dashed lines). Shaded areas represent 95% confidence intervals. In this plot bias is 0.6 kPa (although not significantly different from zero) and LOA_95_ are -7.1 and 8.3 kPa, respectively. Note that the plot shows heteroscedasticity (larger differences to the right than to the left).

**Standard error of the measurement (SEM) and smallest detectable change (SDC)**

The standard error of the measurement (SEM) is used to estimate the measurement error.^6^ SEM may be estimated in several ways.^8^ From SEM, the smallest detectable change (SDC) is determined, representing the range outside which a new measurement would have to lie to be considered caused by a change in the underlying trait at some degree of certainty (often 95%). ^6, 8, 9^

***In the study***

Agreement analysis was performed as described by Bland and Altman. We checked the underlying assumptions of normality (with histogram and QQ-plot) and heteroscedasticity (with Bland-Altman plot and absolute differences plotted vs mean kPa).

In the present study, SEM was determined using the formula:

$$SEM= \frac{{SD}_{d}}{\sqrt{2}}$$

The smallest detectable change (SDC_95_), was derived from SEM by:

$${SDC}_{95}=SEM \times1.96 \times\sqrt{2}$$

This is equivalent to the LOA_95_, but without the bias component.

**RELIABILITY**

Reliability refers to an instrument´s ability to discriminate between subjects.^6, 10^ In contrast to agreement, it is depending on measurement error as well as the heterogeneity of the study population. An instrument with large measurement error may still be good at discriminating between subjects, if the population is heterogenous enough. Reliability statistics are often expressed as a unitless number between 0 (no reliability) and 1 (perfect reliability).^8^ The interpretation of reliability may seem straightforward, as it describes what percentage of variation in the observed score that is attributable to between-subject variation. General rules of thumb have been proposed (e.g., > 0.75 = “good”).^11, 12^ However, these have been thoroughly critisised as the interpretation is situation- and population-dependent.^5, 8, 13^

**Reliability in continuous variables – the intraclass correlation (ICC)**

Many studies use correlation statistics (e.g., Pearson, Spearman) as a measure of reliability, but this is not recommended, for several reasons.^1, 3, 14^ Correlation measures the strength of association. For example, if one instrument consistently gives a result that is twice the value of another, the correlation would be perfect but the instrument useless. To overcome these shortcomings, the intra-class correlation (ICC) is often used.^6, 8, 10^ The ICC has 10 variants, and choosing the right one may be challenging.^4, 11, 13^

**Reliability in categorical variables – Cohen´s Kappa**

For categorised variables, Kappa is used to determine reliability.^15^ A pitfall with categorisation is that values close to cutoffs will be more prone to reclassification. In addition, there may be floor or ceiling effects where extreme values may not be reclassified even with large variations in the underlying continuous variable.

Dichotomous variables are analysed using Cohen´s Kappa.^15^ For categorical variables with more than two categories, weighted Kappa is often used. Kappa weights may be equal (so that the effect on Kappa of a recategorisation by two scale steps is twice that of one step) or squared (so that the effect on Kappa increases more with a larger recategorisation).

***In the study***

Intra-rater reliability was tested by a random-effects single test intra-class correlation. The calculation of ICC starts with a single-factor, within-subjects repeated measures ANOVA (analysis of variance).^8^

For categorical reliability, we used kPa values categorized according to Castera.^16^ For the dichotomous outcome F4 yes vs no we used a regular Kappa estimation. For the ordered categories F0/F1, F2, F3 and F4, we used weighted Kappa with equal weights.

**RESULTS**

**Bland Altman analysis on the original scale**

Plots for the initial Bland Altman analysis on the original scale are shown below:

**Figure S2.** Bland-Altman analysis on the original scale. Histogram and QQ-plot (top) showing suspected non-normality with heavy tails. Correlation plot confirming heteroscedasticity with Kendall´s tau of 0.40 (p <0.001) BA-plot (lower right) is the same as in main manuscript (fig 3A).

| **Measure** | **kPa** |
| --- | --- |
| Mean difference(bias) | 0.6 |
| SD_d_ | 3.9 |
| LOA_95-lower_ | -7.1 |
| LOA_95-upper_ | 8.3 |
| SEM | 2.76 |
| SDC_95_ | ±7.7 |

**Table S1.** Results from Bland-Altman analysis on the original scale. SD = standard deviation, LOA = limits of agreement, SEM = standard error of measurement, SDC = smallest detectable change.

**Interpretation:** As expected, there was no significant systematic bias (95% CI overlapping zero). The LOA_95_ means that if two measurements are taken, they would in 95% of cases be within -7.1 to 8.3 kPa of each other (LOA_95_). The SDC_95_ of ± 7.7 kPa entails that a change of ≥ 7.7 kPa is needed to state with 95% certainty that a change in liver stiffness has occurred between two measurements. However, assumptions were violated, and these results may not be valid.

**Bland Altman analysis - log transformed values**

The remedy for heteroscedasticity, suggested by Bland and Altman, is to use the log transformed values, using the natural logarithm:

**Figure S3.** Bland Altman analysis on the log scale. Histograms and qq-plots look better but one outlier is potentially violation the assumption. Heteroscedasticity has improved. The BA-plot is figure 3B in the main text.

| **Measure** | **kPa** |
| --- | --- |
| Mean difference(bias) | 0.03 |
| SD_d_ | 0.345 |
| LOA_95-lower_ | -0.65 |
| LOA_95-upper_ | 0.70 |
| SEM | 0.244 |
| SDC_95_ | ±0.68 |

**Table S2.** Results from Bland-Altman analysis on the log scale. SD = standard deviation, LOA = limits of agreement, SEM = standard error of measurement, SDC = smallest detectable change.

**Interpretation:** Bias was still nonsignificant. The SDC_95_ was ±0.68, the antilog of 0.68 is 1.97. This means that the SDC_95_ is a ratio of 1.97. Thus, as an example, if one measurement is 8.0 kPa, a new measurement must be below 4.1 kPa (8.0 / 1.97) or above 15.8 kPa (8.0 * 1.97) in order to state that a change has occurred with 95% certainty in the underlying trait. This was the main analysis in the manuscript.

**Agreement - sensitivity analyses**

**Outlier removal**

In the previous analysis, one extreme outlier was found, which may be considered to violate the normality assumption. This outlier was checked for data entry errors, but none were found. Therefore, we performed an analysis where the outlier was removed, to determine the influence of this sole observation. In this analysis log transformed values were used:

| **Measure** | **kPa** |
| --- | --- |
| Mean difference(bias) | -0.05 |
| SD_d_ | 0.302 |
| LOA_95-lower_ | -0.64 |
| LOA_95-upper_ | 0.54 |
| SEM | 0.214 |
| SDC_95_ | ±0.59 |

**Table S3.** Results from Bland-Altman analysis on the log scale, with one outlier removed. SD = standard deviation, LOA = limits of agreement, SEM = standard error of measurement, SDC = smallest detectable change.

**Interpretation**: The antilog of ±0.59 is a ratio of 1.81, quite a bit lower than 1.97 but still substantial. This single outlier did have a disproportionate effect on the results and may have violated the normality assumption, but it was kept in the main analysis.

**Non-parametric Bland - Altman analysis**

Furthermore, a non-parametric version of LOA_95_ was performed, as suggested by Bland and Altman, as a remedy for non-normality.^2^ The non-parametric LOA_95_ was plotted in the same plot as the previous, log-transformed, analysis:

**Figure S4.** Non-parametric Bland Altman plot. Red solid lines represent non-parametric LOA_95_, solid blue line = bias, dashed blue lines = original LOA_95_, shaded areas = 95% confidence intervals of original plot.

**Interpretation:** The non-parametric analysis gave a slightly narrower LOA_95_ but did not differ significantly from the original analysis, which was kept as the main analysis.

**Changing the SDC level of certainty**

In addition to the SDC_95_ in the main analysis, we estimated SDC_90_ and SDC_80_ using the logged measurements to determine the smallest detectable change with 90% and 80% certainty, respectively. The constant was changed from 1.96 for the SDC_95_ to 1.645 and 1.282 for SDC_90_ and SDC_80_. This resulted in SDC_90_ = 0.57 (antilog 1.76) and SDC_80_ = 0.44 (antilog 1.56).

**Interpretation:** Changing the level of certainty decreased the SDC as expected. To say that a change has occurred with 90% certainty with a first measure of 8.0 kPa, a new measure would have to be below 4.5 kPa or above 14.1 kPa (compared to 4.1 and 15.8 in the main analysis).

***Post-hoc* analysis**

As fasting time was associated with differences ≥ 33% in bivariate analysis, we plotted differences vs fasting time. In this plot it was clear that participants with fasting time less than five hours had a larger variability.

**Figure S5.** Inter-rater differences vs fasting. Each dot represents one patient, with the inter-rater differences in kPa on the y axis and the time since food intake in hours on the x axis.

| **Operator** | **Years of TE experience*** | **Title** |
| --- | --- | --- |
| OL | >3 | M.D. Specialist in infectious diseases |
| CK | >3 | M.D. Specialist in infectious diseases |
| JO | >3 | M.D. Specialist in infectious diseases |
| JS | >3 | M.D. Specialist in infectious diseases |
| AS | >3 | M.D. Specialist in infectious diseases |
| JB | >3 | M.D. Specialist in infectious diseases |
| JT | <3 | M.D. Resident physicians |
| ACL | <3 | M.D. Resident physicians |
| CS | >3 | Nurse. Specially trained in TE. |

**Table S4.** The TE experience of each observer. *at the beginning of the study. M.D. = medical doctor

References

1. Bland JM, Altman DG. Statistical methods for assessing agreement between two methods of clinical measurement. *Lancet*. **1986**;1(8476):307-10.

2. Bland JM, Altman DG. Measuring agreement in method comparison studies. *Stat Methods Med Res*. **1999**;8(2):135-60.

3. Bland JM, Altman DG. Applying the right statistics: analyses of measurement studies. *Ultrasound Obstet Gynecol*. **2003**;22(1):85-93.

4. Hernaez R. Reliability and agreement studies: a guide for clinical investigators. *Gut*. **2015**;64(7):1018-27.

5. Bland JM, Altman DG. A note on the use of the intraclass correlation coefficient in the evaluation of agreement between two methods of measurement. *Comput Biol Med*. **1990**;20(5):337-40.

6. de Vet HC, Terwee CB, Knol DL, Bouter LM. When to use agreement versus reliability measures. *J Clin Epidemiol*. **2006**;59(10):1033-9.

7. Kottner J, Streiner DL. The difference between reliability and agreement. *J Clin Epidemiol*. **2011**;64(6):701-2; author reply 2.

8. Weir JP. Quantifying test-retest reliability using the intraclass correlation coefficient and the SEM. *J Strength Cond Res*. **2005**;19(1):231-40.

9. Bartlett JW, Frost C. Reliability, repeatability and reproducibility: analysis of measurement errors in continuous variables. *Ultrasound Obstet Gynecol*. **2008**;31(4):466-75.

10. Kottner J, Audigé L, Brorson S, Donner A, Gajewski BJ, Hróbjartsson A, et al. Guidelines for Reporting Reliability and Agreement Studies (GRRAS) were proposed. *J Clin Epidemiol*. **2011**;64(1):96-106.

11. Koo TK, Li MY. A Guideline of Selecting and Reporting Intraclass Correlation Coefficients for Reliability Research. *J Chiropr Med*. **2016**;15(2):155-63.

12. Lee J, Koh D, Ong CN. Statistical evaluation of agreement between two methods for measuring a quantitative variable. *Comput Biol Med*. **1989**;19(1):61-70.

13. Liljequist D, Elfving B, Skavberg Roaldsen K. Intraclass correlation - A discussion and demonstration of basic features. *PLoS One*. **2019**;14(7):e0219854.

14. Bunting KV, Steeds RP, Slater LT, Rogers JK, Gkoutos GV, Kotecha D. A Practical Guide to Assess the Reproducibility of Echocardiographic Measurements. *J Am Soc Echocardiogr*. **2019**;32(12):1505-15.

15. McHugh ML. Interrater reliability: the kappa statistic. *Biochem Med (Zagreb)*. **2012**;22(3):276-82.

16. Castera L. Transient elastography and other noninvasive tests to assess hepatic fibrosis in patients with viral hepatitis. *J Viral Hepat*. **2009**;16(5):300-14.
